# Identification of the defense-related gene *VdWRKY53* from the wild grapevine *Vitis davidii* using RNA sequencing and ectopic expression analysis in Arabidopsis

**DOI:** 10.1186/s41065-019-0089-5

**Published:** 2019-04-26

**Authors:** Ying Zhang, Jia-Long Yao, Hu Feng, Jianfu Jiang, Xiucai Fan, Yun-Fei Jia, Ran Wang, Chonghuai Liu

**Affiliations:** 1 Zhengzhou Fruit Research Institute, Chinese Academy of Agriculture Sciences, Zhengzhou, 450009 China; 2 The New Zealand Institute for Plant & Food Research Limited, Auckland, New Zealand

**Keywords:** Chinese wild grapevine, Disease resistance, Grapevine white rot, Transcriptome, VdWRKY53 transcription factor

## Abstract

**Background:**

Grapevine is an important fruit crop grown worldwide, and its cultivars are mostly derived from the European species *Vitis vinifera*, which has genes for high fruit quality and adaptation to a wide variety of climatic conditions. Disease resistance varies substantially across grapevine species; however, the molecular mechanisms underlying such variation remain uncharacterized.

**Results:**

The anatomical structure and disease symptoms of grapevine leaves were analyzed for two grapevine species, and the critical period of resistance of grapevine to pathogenic bacteria was determined to be 12 h post inoculation (hpi). Differentially expressed genes (DEGs) were identified from transcriptome analysis of leaf samples obtained at 12 and 36 hpi, and the transcripts in four pathways (cell wall genes, LRR receptor-like genes, *WRKY* genes, and pathogenesis-related (PR) genes) were classified into four co-expression groups by using weighted correlation network analysis (WGCNA). The gene *VdWRKY53*, showing the highest transcript level, was introduced into Arabidopsis plants by using a vector containing the CaMV35S promoter. These procedures allowed identifying the key genes contributing to differences in disease resistance between a strongly resistant accession of a wild grapevine species *Vitis davidii* (*VID*) and a susceptible cultivar of *V. vinifera*, ‘Manicure Finger’ (*VIV*). *Vitis davidii,* but not *VIV,* showed a typical hypersensitive response after infection with a fungal pathogen (*Coniella diplodiella*) causing white rot disease. Further, 20 defense-related genes were identified, and their differential expression between the two grapevine species was confirmed using quantitative real-time PCR analysis. *VdWRKY53*, showing the highest transcript level, was selected for functional analysis and therefore over-expressed in Arabidopsis under the control of the CaMV35S promoter. The transgenic plants showed enhanced resistance to *C. diplodiella* and to two other pathogens, *Pseudomonas syringae* pv. *tomato* DC3000 and *Golovinomyces cichoracearum*.

**Conclusion:**

The consistency of the results in *VID* and transgenic Arabidopsis indicated that *VdWRKY53* might be involved in the activation of defense-related genes that enhance the resistance of these plants to pathogens. Thus, the over-expression of *VdWRKY53* in transgenic grapevines might improve their resistance to pathogens.

**Electronic supplementary material:**

The online version of this article (10.1186/s41065-019-0089-5) contains supplementary material, which is available to authorized users.

## Background

Grapevine is an important fruit crop grown worldwide, and its cultivars are mostly derived from the European species *Vitis vinifera*, which has genes for high fruit quality and adaptation to a wide variety of climatic conditions. However, *V. vinifera* cultivars are susceptible to many pathogens such as phytoplasmas, viruses, bacteria, oomycetes, and fungi [1].

The fungus *Coniella diplodiella* (Speg.) Petr. & Syd., which belongs to family Schizoparmaceae, causes the devastating white rot disease on grape berries at the ripening stage, resulting in partial to total crop losses. This disease also has severe impacts on the environment because repeated fungicide applications are required to control it. White rot disease is a main grapevine disease not only in China but also in other grapevine-growing regions worldwide. The disease symptoms appear primarily on the fruit tissues as well as on stems and leaves from 3 to 6 days after the infection [2]. *Coniella diplodiella* obtains nutrients from infected tissues and eventually decomposes these tissues; however, the molecular mechanism underlying the defense against *C. diplodiella* is not yet clear. We found that WRKY gene family members were induced in grapevines under white rot and salicylic acid (SA) stress, and 16 WRKY genes were upregulated both by white rot pathogenic bacteria and SA; however, the resistance mechanism to *C. diplodiella* remained unknown [3].

The WRKY transcription factors comprise a large family of regulatory proteins and have been implicated in the defense against pathogens in plants [4]. The over-expression of two grapevine WRKY genes, *VvWRKY1* and *VvWRKY2,* conferred enhanced resistance against fungal pathogens in transgenic tobacco plants [5]. *VvWRKY53* may play a role in eliciting resistance response during the early stage of infection by powdery mildew. It appears to share similar inoculation response with *VvWRKY1*, as reported previously [6, 7], whereas knockout of *VvWRKY52* increased grapevine resistance to *Botrytis cinerea* [8]. *VvWRKY33* can confer resistance to oomycete pathogens when it transiently expressed in leaves [9]. The ectopic expression of *VvWRKY11* provides higher tolerance to water stress induced by mannitol in Arabidopsis [10]. The *VpWRKY1*, *VpWRKY2*, *VpWRKY3,* and *VlWRKY48* and *VlWRKY3* genes isolated from *V. pseudoreticulata* and *V. labrusca* × *V. vinifera* cv. ‘Kyoho’, respectively, enhanced the resistance of these grapevines to biotic and abiotic stresses [11–15]. The *AtWRKY53* gene of Arabidopsis, orthologous to *VvWRKY53*, was rapidly induced under drought stress [14] and positively regulated basal resistance to *Pseudomonas syringae* pv. *tomato* DC3000 (*PstDC3000*) in combination with *AtWRKY46* and *AtWRKY70* [15]. The tomato gene orthologous to *AtWRKY53*, *SlWRKY53*, confers resistance to tomato yellow leaf curl virus [16].

To ward off tenacious pathogens, plants have developed a vast array of immune responses. Plants, including *Vitis* species, exhibit different resistance levels depending on their different immune mechanisms. In contrast to *V. vinifera* cultivars, clones of wild grapevine species exhibit high levels of resistance [2] and are used as parental plants to map the major quantitative trait loci of resistance genes [17, 18]. Breeders can introgress these resistance genes into *V. vinifera*-based cultivars, and conventional breeding has led to the development of some interspecific hybrids that are resistant to fungal diseases. However, this process is long and inefficient. Further breeding programs are required to develop disease-resistant table and wine grapevines that also have high grape berry quality. Such breeding processes might be remarkably accelerated by the availability of grapevine genome sequences [19] and marker-assisted selection [20].

Of the approximately 70 *Vitis* species worldwide, 38 have originated in China [21]. Chinese wild grapevines are very important germplasms for breeding new cultivars as they possess resistance genes and special resistance mechanisms [22]. We collected 500 accessions from 20 Chinese wild grapevine species and maintained them in a germplasm repository. From this collection, the accession *Vitis davidii* 0940 showed the highest level of resistance to white rot disease in in vitro analyses [23]. However, the genes associated with the resistance trait have not yet been identified in this grapevine accession. Resistance genes are often induced by pathogen infection in resistant plants [24, 25]. The expression of these genes might be considerably weaker or even absent in susceptible plants. Resistance genes can be identified as differentially expressed genes (DEGs) by conducting comparative analysis of the transcriptomes of resistant and susceptible plants after infection.

In this study, we aimed to identify candidate resistance genes in *Vitis davidii* by comparing its transcriptome with that of *V. vinifera*. We found that the expression of 20 defense-related genes was induced in *V. davidii*, but not in *V. vinifera* tissues at the early stage of *C. diplodiella* infection. One of these genes, *VdWRKY53,* was confirmed to confer resistance to a wide range of pathogens, including *C. diplodiella*, *PstDC3000*, and *Golovinomyces cichoracearum* (powdery mildew of Arabidopsis) when it was over-expressed in transgenic Arabidopsis plants.

## Methods

### Observation of the microstructure and ultrastructure of grapevine leaves

Leaf tissues (0.25 cm^2^) were collected from the middle of five healthy mature leaves from *V. vinifera* ‘Manicure Finger’ (*VIV*) and *V. davidii* accession 0940 (*VID*) and fixed in formalin-acetic acid-alcohol fixative. The samples were then embedded in paraffin and cut into 10-μm-thick sections, stained with hematoxylin, examined under a Leica DMi1-PH1 microscope (Leica Camera AG, Wetzlar, Germany), and photographed using an Olympus BX51 camera (Olympus Corp., Tokyo, Japan). Leaf ultrastructure was observed, and images were captured using a HITACHI 7000 (Hitachi Ltd., Tokyo, Japan) transmission electron microscope (TEM) by using the method described by Lighezan et al. [26].

### Trypan blue staining

To visualize microscopic lesions, leaves were stained using the method described by Vogel and Somerville [27] with slight modification. Grape leaves were cut into 1 cm^2^ segments, placed in 50-mL conical bottles, and vacuum-infiltrated twice in a solution of phenol, lactic acid, glycerol, and water (1:1:1:1) containing 250 mg/mL trypan blue. The conical bottles were then placed in a boiling water bath for 2 min and allowed to cool for 1 h. The leaves were de-stained in the 1:1:1:1 solution for 1 h and examined under bright-field microscopy.

### Plant materials and pathogen inoculation treatments on grapevine

For RNA-sequencing (RNA-seq) analysis, 2-year-old plants of *VIV* and *VID* were grown in a greenhouse at 28 °C with a 16-h photoperiod. The plants were inoculated with *C. diplodiella* (strain WR01, from the Institute of Plant Protection, Chinese Academy of Agricultural Sciences) by fixing four mycelium gelose discs (diameter = 2 mm) on each leaf using small pins, and covering each leaf with a plastic bag to maintain the moisture of the gelose disks during the entire infection period. The mycelium gelose discs were prepared from a *C. diplodiella* culture grown at 28 °C on potato dextrose agar medium. Leaf samples (plugged around the infection spot) were collected at 0, 12, and 36 h post inoculation (hpi); each treatment had three independent biological replicates and three mock-infected replicates. The samples were immediately frozen in liquid nitrogen and stored at − 80 °C. Each sample consisted of pooled specimens from the replicates within each treatment.

### RNA extraction, library construction, and RNA-seq

For RNA-seq library construction, total RNA was extracted from three biological replicates and three mocks at one time point by using the Total RNA Extraction Kit (BioFlux, Tokyo, Japan). The infected samples named *VIV1*, *VIV2*, *VIV3*, for *VIV* and *VID1*, *VID2,* and *VID3* for *VID* were collected at 0, 12, and 36 hpi. The RNA quality and purity were checked on 1% agarose gels by using a NanoPhotometer® spectrophotometer (Implen GmbH, München, Germany).

Sequencing libraries were generated using 3 μg of RNA per sample as input material and the NEB Next® Ultra™ RNA Library Prep Kit (NEB, Ipswich, MA, USA) for Illumina sequencing (Illumina Inc., San Diego, CA, USA). Index codes were added to each sample to tag the sequences. The index-coded samples were clustered on a cBot Cluster Generation System by using the TruSeq PE Cluster Kit v3-cBot-HS (Illumina), following the manufacturer’s instructions. After the clusters were generated, the libraries were sequenced by Novogene (Beijing, China) on an Illumina HiSeq 2000 platform to generate 100 bp paired-end reads.

### Data assembly and analyses

Clean data (clean reads) were obtained by removing the adapter sequences in the reads. Simultaneously, the Q20, Q30, and GC contents of the clean reads were calculated. All downstream analyses were performed using high-quality clean data.

#### Reference genome and gene model annotation files were downloaded from the genome website (http://plants.ensembl.org/Vitis_vinifera). An index of the reference genome was generated using bowtie v2.0.6 (https://sourceforge.net/projects/bowtie-bio/files/bowtie2/2.1.0/) and paired-end clean reads were aligned to the reference genome by using TopHat v2.0.9 (https://ccb.jhu.edu/software/tophat/index.shtml). The number of reads mapped to each gene was counted using HTSeq v0.5.4p3 (https://pypi.python.org/pypi/HTSeq/0.5.4p3). In addition, the reads per kilobase of transcript per million mapped reads (RPKM) of each gene was calculated based on the length of the gene transcript, the reads mapped to the gene, and the total number of mapped reads [28]

The differential expression of each gene under two conditions (infected and non-infected) was analyzed using the DEGseq R package (1.12.0) [29]. The data were analyzed by subtracting each treatment dataset from the corresponding mock dataset. A corrected *P*-value of 0.001 and a log_2_ (fold change) of 1 were set as the thresholds for determining significant differentially expressed genes (DEGs).

### Gene co-expression analysis

Gene co-expression network analysis was performed for each RNA-seq library to identify genes with similar expression patterns in each experimental sample, according to the methods described by J Gillis, followed by a search for resistance-related pathways and genes [30]. The weighted correlation network analysis method was also used for detecting clusters (modules) of highly correlated genes [31]. We subjected the best WGCNA results to MeV *K*-means analysis (http://www.tm4.org/mev.html) by setting the cluster number to 50 (K = 50).

### Annotation and functional classification

Gene Ontology (GO) enrichment analysis of the DEGs was implemented using the GO seq R package (http://www.bioconductor.org/packages/release/bioc/html/goseq.html), in which gene length bias was corrected. The GO terms with corrected *P*-values < 0.05 were considered significantly enriched in the DEGs. The KOBAS software (http://kobas.cbi.pku.edu.cn/) was used to test the statistical enrichment of the DEGs in the Kyoto Encyclopedia of Genes and Genomes (KEGG) pathways. Putative gene functions were assigned using a set of sequential basic local alignment search tool (BLAST) searches of all the assembled unigenes against sequences in the Ensembl Plants (http://plants.ensembl.org/Vitis_vinifera) database of non-redundant proteins and nucleotides (Nr), the Swiss-Prot protein (UniProt) database, the GO database, the Cluster of Orthologous Groups (COGs) database, and the KEGG database.

The full-length amino acid sequence (listed in Additiopnal file S4) and neighbor joining (NJ) method in Clustal X version 1.83 and MEGA version 5.0 (https://mega.software.informer.com/5.0/) were used.

### Quantitative real-time PCR

Total RNA samples of grapevines and Arabidopsis were extracted using the improved sodium dodecyl sulfate/phenol method described by Ulker [32]. The PCR primers used for the reference genes (*EF1r* in grapevine and *AtSAND* At2g28390 in Arabidopsis) [33] and the test genes are listed in Additional file 1: Table S1. The PCRs were conducted using three biological and three technical replicates for each gene in a LightCycler® 480 (Roche Diagnostics, Basel, Switzerland). The relative expression levels of the genes were calculated using the 2^-△△ct^ method [34, 35].

### Vector construction and Arabidopsis transformation

The full-length cDNA of *VdWRKY53* was amplified using PCR and cloned into the *BglII/BstE2* site of the 35S promoter in a sense orientation in the binary plasmid pCAMBIA1301 to form the plasmid pCAMBIA1301-VdWRKY53 (pGW53). This new plasmid was verified by sequencing and then introduced into *Agrobacterium tumefaciens* GV3101 cells for Arabidopsis transformation through the floral dipping method [36, 37].

### Pathogenic fungus/bacterium inoculation of Arabidopsis

Homozygous transgenic Arabidopsis plants were used for pathogen inoculation. They were identified from three independent transgenic lines by using hygromycin antibiotic selection and grown to the T3 generation. If 100% of the T3 plants were hygromycin resistant, the T2 plants were considered as homozygous transgenic plants, and their seeds were re-sown directly into soil without hygromycin selection to generate T3 plants for pathogen inoculation. *Coniella diplodiella* was inoculated on wild-type (Columbia, Col) and T3 homozygous transgenic (GW53) Arabidopsis plants grown in a chamber at 25 °C with a 12-h photoperiod and light intensity of 100 μmol m^− 2^ s^− 1^. The powdery mildew (*G. cichoracearum*) isolate UCSC1 was cultured on the Arabidopsis *phytoalexin cichoracent 4* (*pad4*) mutant plants. Powdery mildew inoculation of Arabidopsis was performed as previously described [38]. The bacterial strain *PstDC3000* was grown in Luria-Bertani liquid medium [39] and inoculated by using the method of Melotto et al. [40].

## Results

### Anatomical structure and disease symptoms of grapevine leaves

**Fig. 1:**
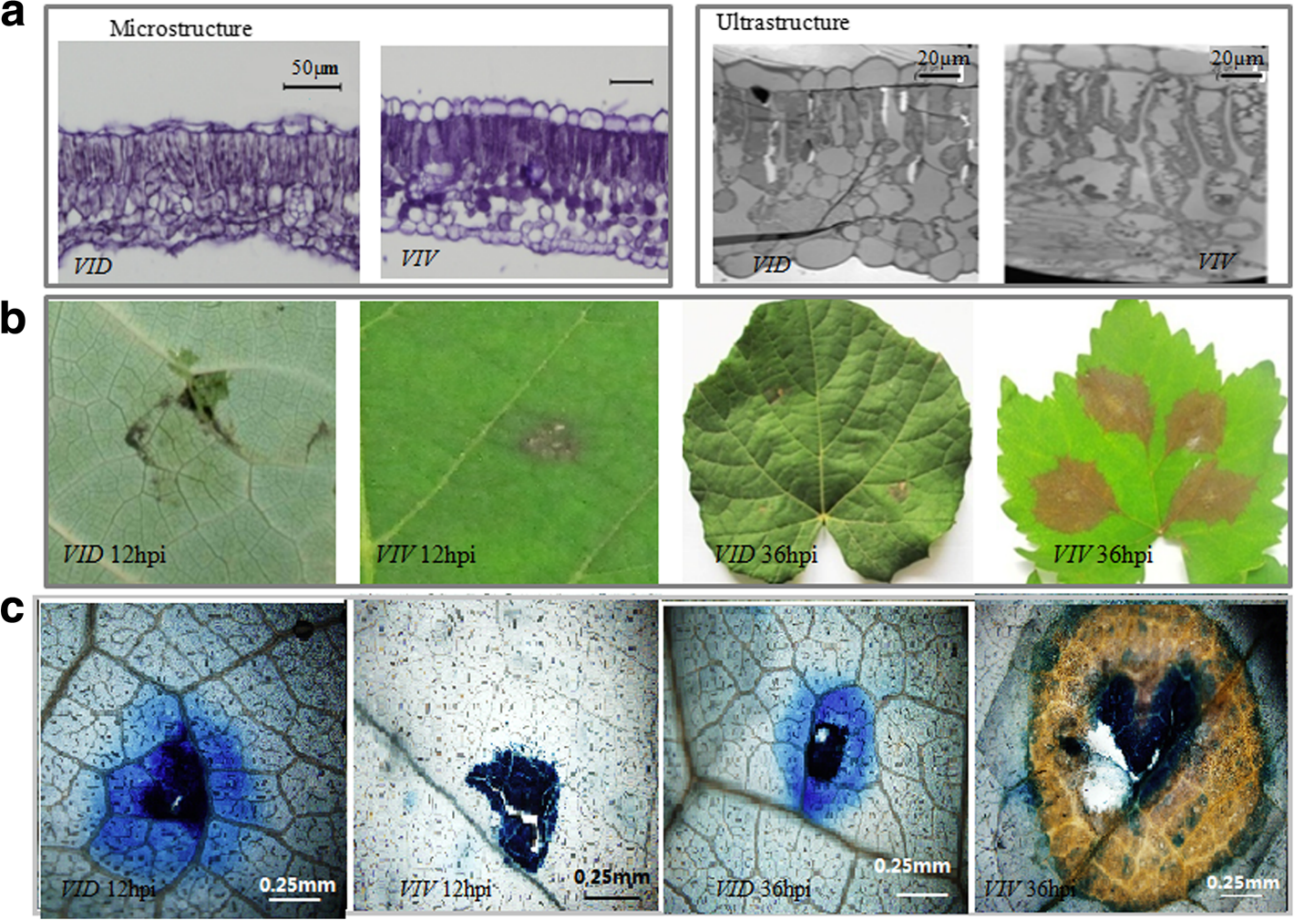
*Vitis* leaf structure and symptoms of *Coniella diplodiella* infection. **a** Anatomical structure of *Vitis vinifera* cv. Manicure Finger (*VIV*) and *Vitis davidii* accession 0940 (*VID*). Leaf samples were collected from 2-week-old leaves at 3–4 positions on a branch. At the microstructure level, the leaf thickness, upper epidermis thickness, palisade tissue thickness, spongy tissue thickness, and lower epidermis thickness were not significantly different between *VID* and *VIV*. At the ultrastructure level, *VID* had more chloroplasts in the palisade tissue than in *VIV*. **b** Symptoms in *VID* and *VIV* after *C. diplodiella* infection. Typical hypersensitive response (HR) symptoms were observed in *VID*, but not in *VIV* at 12 h post-infection (hpi) and 36 hpi

*Vitis davidii* is an important wild grapevine species that grows in 10 provinces of China. In our previous studies, *VID* showed the highest level of resistance among all grapevines tested [23, 41]. In the present study, anatomical structure analysis revealed that the leaves of *VID* were not significantly different from those of *VIV* in thickness, including the thickness of the palisade, spongy tissues, and upper and lower epidermis (Table 1, Fig. 1a). Except for the higher number of chloroplasts in the palisade of *VID* than in the palisade of *VIV* (Fig. 1a), which is important for photosynthesis, *VID* had a similar leaf structure to *VIV*. This result suggested that the differences in disease resistance between *VID* and *VIV* are likely related to genetic factors and not to anatomic features.

**Table 1:** Comparison of leaf tissue thickness between the two *Vitis* species

Species	Leaf thickness (μm)	Upper epidermis thickness (μm)	Palisade tissue thickness (μm)	Spongy tissue thickness (μm)	Lower epidermis thickness (μm)
*Vitis davidii*	91.13 ± 7.48^a^	12.43 ± 2.15^a^	36.44 ± 4.32^a^	36.33 ± 7.70^a^	7.87 ± 1.72^a^
*Vitis vinifera*	112.78 ± 18.50^a^	12.56 ± 2.82^a^	42.75 ± 8.53^a^	48.98 ± 8.49^a^	9.43 ± 1.84^a^

Data are the mean ± SD, *n* = 12. Significant differences were assessed using analysis of variance

After infection with *C. diplodiella*, the disease symptoms were weaker on *VID* leaves than on *VIV* leaves at 12 hpi. After trypan blue staining, at 12 and 36 hpi, the symptoms on *VID* leaves developed into a typical hypersensitive response (HR), showing cell death at the infection site and limited spreading of the pathogen, but the symptoms on *VIV* leaves developed into typical grapevine white rot disease (Fig. 1b, c), with no HR cell death. Considering that disease development occurs between 12 and 36 hpi, we selected these two time points for transcriptome comparison with that at 0 hpi.

### RNA sequencing analysis

Approximately 433 million reads were generated from the six libraries (*VIV1*, *VIV2*, *VIV3*, *VID1*, *VID2*, and *VID3*), prepared from the leaf tissues collected at 0, 12, and 36 hpi for comparing the transcript levels of *VID* and *VIV* in response to *C. diplodiella* infection. These reads contained 42.32 Giga-bases (Gb) of cDNA (Table 2). More than 96% of these reads had high quality (Q > 20) and, therefore, they were selected for further analyses. Of the clean reads, 77–88% were mapped to the *V. vinifera* reference genome (http://plants.ensembl.org/Vitis_vinifera) [20]. For each library, the reads were mapped to approximately 23,000–25,000 genes, of which approximately 1000 were novel genes that were not annotated in the grapevine reference genome (Table 2).

**Table 2:** Summary of the mean transcriptome data

	*VIV1*	*VIV2*	*VIV3*	*VID1*	*VID2*	*VID3*
Total reads (× 1000)	76,192	76,999	67,696	67,447	71,886	73,179
Base number (Gb)	7.62	6.7	6.76	6.74	7.18	7.32
High-quality reads (%)	98.1	96.48	97.97	96.31	98.02	96.46
Mapped reads (%)	88.01	87.27	87.43	77.33	76.96	77.14
Number of transcripts	23,621	24,301	24,407	23,441	25,537	24,018
Number of novel transcripts	961	971	997	938	1020	957

### Identification of DEGs

According to the DEGseq, 12,976 genes had RPKM > 1 and were, therefore, selected for DEG analysis. The number of DEGs between the resistant (*VID*) and susceptible (*VIV*) grapevine plants were analyzed at each time point after *C. diplodiella* inoculation.

**Fig. 2:**
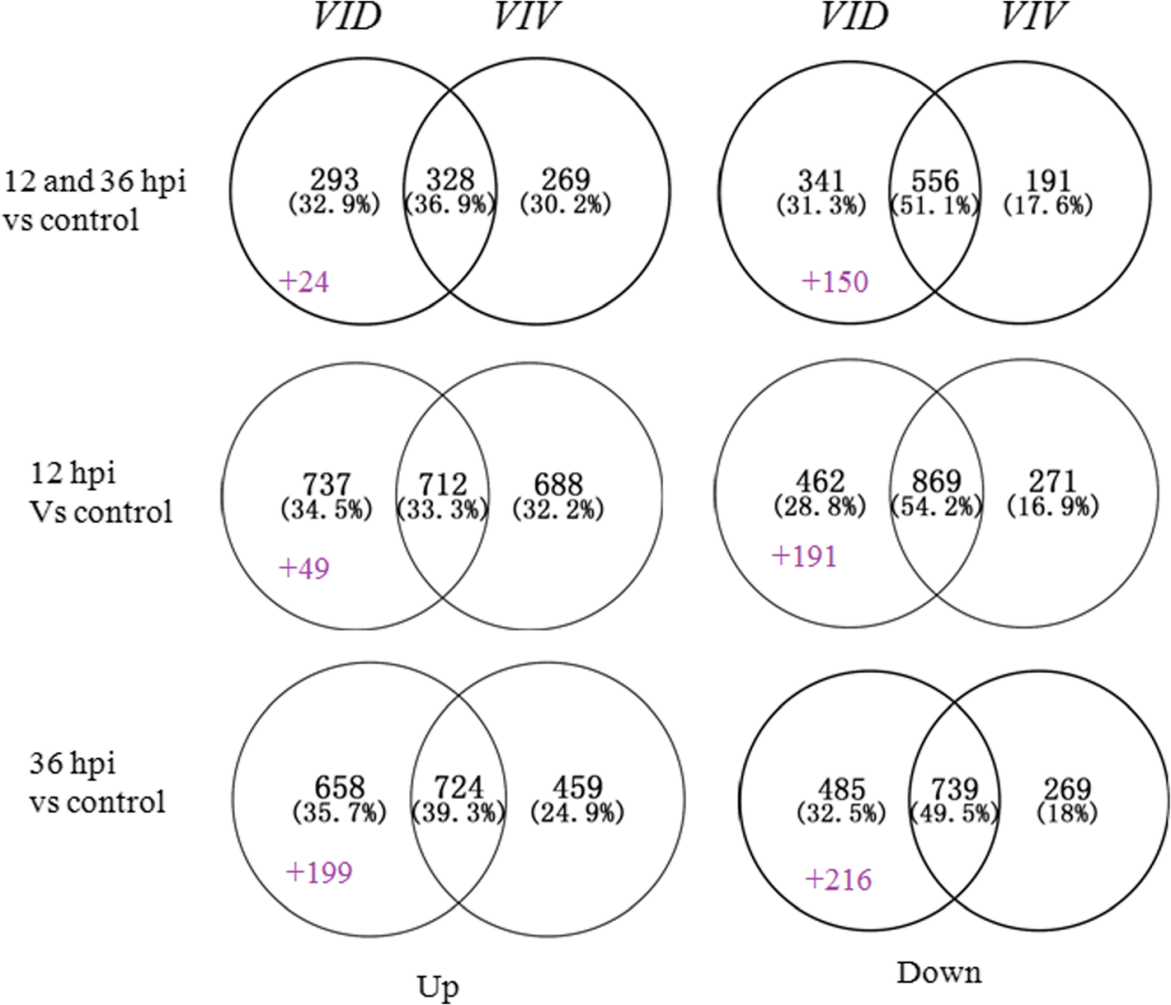
Number of differentially expressed genes in *Vitis davidii* accession 0940 (*VID*) and *Vitis vinifera* cv. Manicure Finger (*VIV*) leaves following infection with *Coniella diplodiella*. Venn diagrams show the number of differentially expressed genes (DEGs), up- or down-regulated (*P* < 0.0001, > 2.0-fold), in *VID* and *VIV* leaves at 12 and 36 h post-infection (hpi) compared to those at 0 hpi

A total of 7073 transcripts, differing in expression between the two grapevines, were induced. Two hundred and fifty-six transcripts specifically expressed in *VID* were from novel genes that were not identified in the reference genome (http://plants.ensembl.org/Vitis_vinifera). This could be due to the large genetic distance between *VID* and the grapevine species used to generate the reference genome. Overall, more DEGs were found in the resistant genotype *VID* than in the susceptible genotype *VIV*. One-hundred and seventy-four DEGs (24 up-regulated and 150 down-regulated) were more expressed in *VID* than in *VIV* at 12 and 36 hpi, while the expression of 240 more DEGs (49 up-regulated and 191 down-regulated) changed more in *VID* than in *VIV* at 12 hpi, and that of 415 DEGs (199 up-regulated and 216 down-regulated) changed more in *VID* than in *VIV* at 36 hpi (Fig. 2).

All DEGs were annotated by searching multiple databases, including COG, GO, KEGG, Swiss-Prot, and nr (Table 3) [42–44]. To determine pathways containing DEGs between the susceptible *VIV* and resistant *VID*, we analyzed the KEGG pathways in detail. We detected DEGs in 118 pathways, but two pathways (brassinosteroid biosynthesis, and cutin, suberine, and wax biosynthesis) contained no DEGs. Differences in gene expression at two time points after pathogen infection in *VIV* and *VID* were examined, and DEGs were identified by pairwise comparisons of the six treatments (Additional file 2: Table S2 and Additional file 3: Table S3).

**Table 3:** Annotation of the differentially expressed genes (DEGs) from six treatments

DEG Set	Annotated	COG	GO	KEGG	Swiss-Prot	nr
*VID2*_vs_*VID1*	3952	1617	3517	675	2997	3952
*VID3*_vs_*VID1*	4025	1657	3594	677	3114	4025
*VIV2*_vs_*VIV1*	3925	1664	3506	656	3031	3925
*VIV3*_vs_*VIV1*	3869	1572	3455	636	2986	3869

**Fig. 3:**
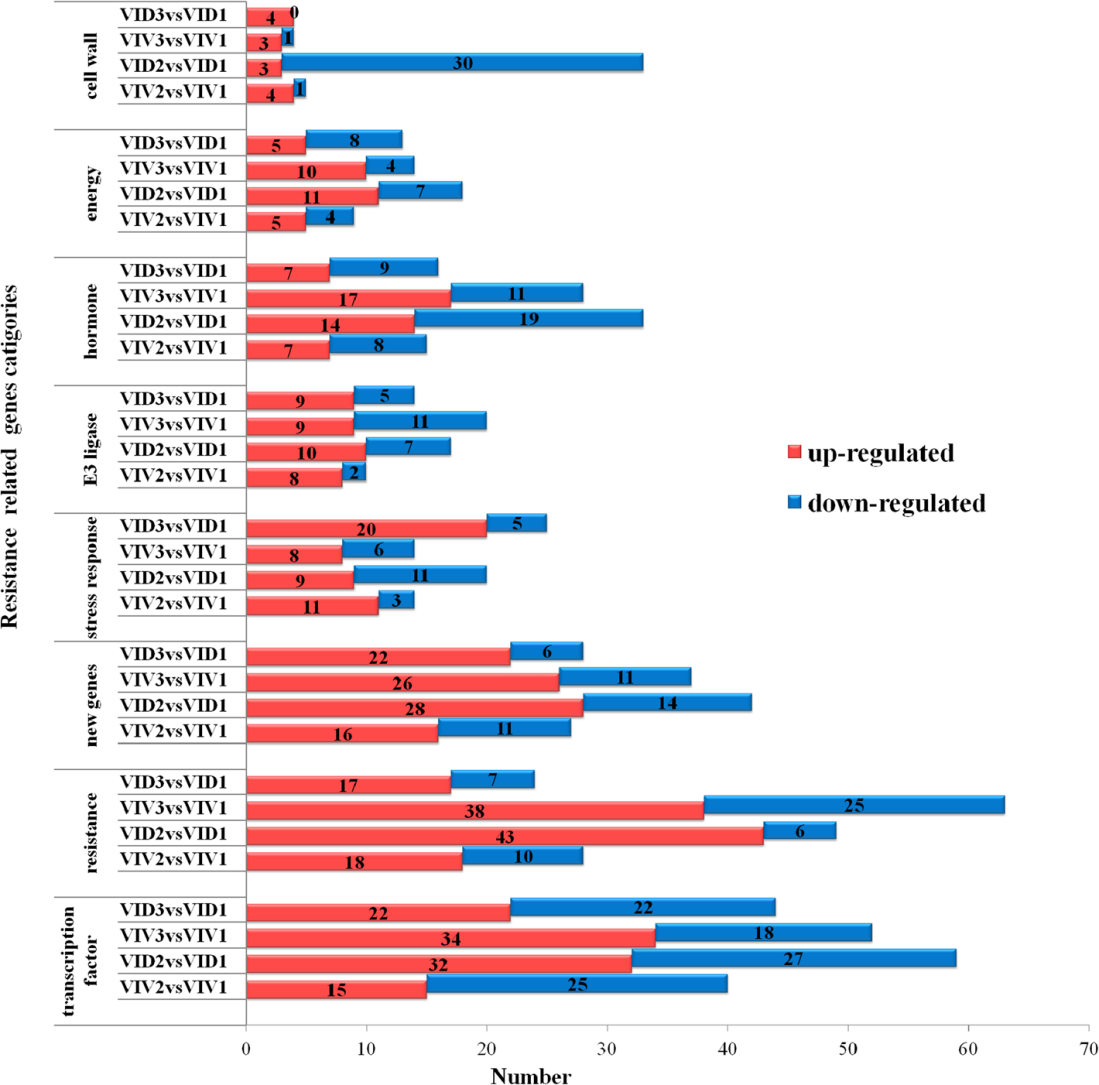
Important function of differentially expressed genes in *Vitis davidii* accession 0940 (*VID*) and *Vitis vinifera* cv. Manicure Finger (*VIV*) following infection with *Coniella diplodiella*

We statistically analyzed the number of important functional genes that were induced, including genes related to cell wall, energy, hormone, E3, RNA, transcription factor, and amino acid biosynthesis, stress response, growth, resistance, and basic functions, and new genes (Fig. 3). Among these, 374 genes related to cell wall, energy, hormone, and transcription factor biosynthesis and resistance were induced in *VID2* but not in *VIV2*. In contrast, 28 genes involved in the same processes were induced in *VIV3* but not in *VID3*.

### Identification of candidate resistant genes

**Fig. 4:**
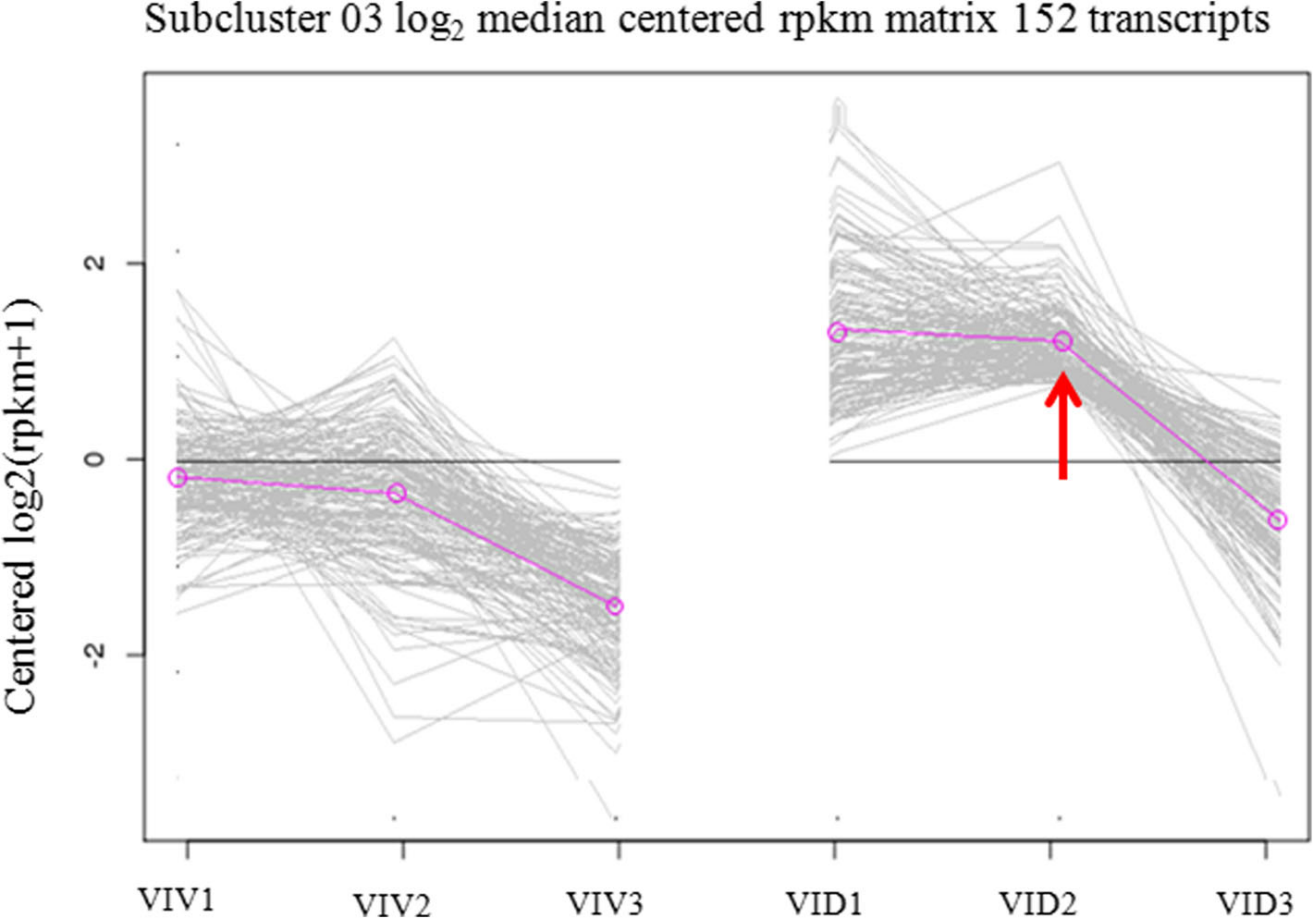
The subcluster K03 from *K*-means co-expression analysis. All differentially expressed genes (DEGs) were divided into 50 co-expression subclusters (K = 50) by using the MeV *K*-means analysis. In subcluster K03, 152 DEGs showed significant co-expression tendency at 12 h post-infection

We focused on plant-pathogen interaction pathways to identify candidate resistant genes. These pathways included cell wall genes, leucine-rich repeat (LRR) receptor-like genes, *WRKY* genes, and pathogenesis-related (PR) genes. All transcripts in these pathways were classified into four co-expression groups by using WGCNA. One of these groups was further divided into 50 co-expression subclusters (K = 50), by using software MeV in *K*-means analysis (http://www.tm4.org/), based on the molecular function and RPKM value of the genes. In one of these subclusters, K03, the transcript levels of 152 genes were higher in *VID* than in *VIV* at 0 and 12 hpi, whereas their transcript levels were reduced at 36 hpi compared to those at 0 and 36 hpi in both *VIV* and *VID* (Fig. 4, Additional file 4: Table S4).

**Fig. 5:**
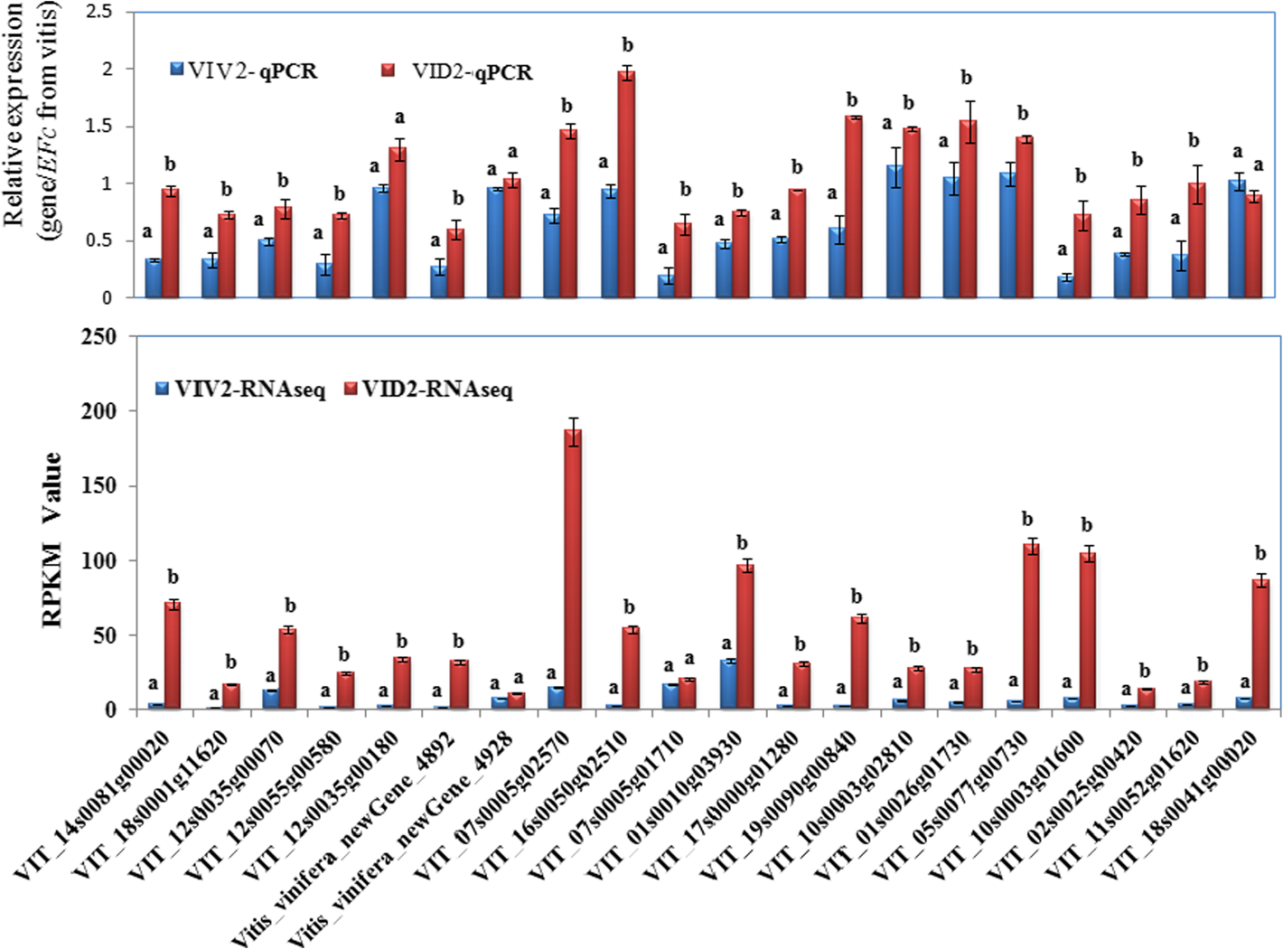
Comparison between RNA sequencing (RNA-seq) and quantitative real-time PCR (qRT-PCR) results for 20 defense-related candidate genes. Based on RNA-seq analysis, 152 DEGs were clustered in K03. Twenty defense-related genes in K03 that showed significant differential expression at 12 h post-infection were further analyzed using qRT-PCR

From subcluster K03, 20 candidate resistant genes from the SA signaling pathway were selected because their expression pattern was highly correlated with the resistance response. Their expression levels in the six libraries obtained for the two grapevine species are listed in Table 4. The selected candidate genes showed RPKM > 1 and log_2_ (fold change) > 2. These 20 genes included two *wall-associated receptor kinase* genes, five *LRR receptor-like serine/threonine-protein kinase* genes, eleven *WRKY transcription factor* genes, and two *PR protein-like* genes. The expression pattern of these candidate genes was confirmed by qRT-PCR analyses by using the RNA extracted at 12 hpi. Nineteen genes that showed a higher RPKM value in *VID* than in *VIV* were confirmed to have higher expression level in *VID* than in *VIV* at 12 hpi*.* Only two genes (*VIT_18s0041g00020* and *VIT_07s0005g01710*) showed an opposite trend between RNA-seq and qRT-PCR analyses (Fig. 5)*.*

**Table 4:** Defense-related candidate genes classified in the co-expression subcluster K03

Gene ID	Gene function		RPKM value				
		VIV1	DA1	VIV2	DA2	VIV3	DA3
VIT_18s0041g00020	Wall-associated receptor kinase 2	9.69	22.35	13.35	16.12	10.79	76.36
VIT_18s0001g11620	Wall-associated receptor kinase 2	0.42	5.24	2.48	19.85	0.60	1.52
VIT_12s0035g00070	LRR receptor-like serine/ threonine-protein kinase	1.55	16.89	2.86	53.64	1.57	54.97
VIT_12s0055g00580	LRR receptor-like serine/threonine-protein kinase	1.35	5.42	2.55	24.08	1.84	5.52
VIT_12s0035g00180	LRR receptor-like serine/threonine-protein kinase	0.75	1.25	0.81	33.93	1.07	2.52
Vitis_vinifera_newGene_4892	LRR receptor-like serine/threonine-protein kinase	0.90	1.79	0.24	8.41	0.05	1.22
Vitis_vinifera_newGene_4928	LRR receptor-like serine/threonine-protein kinase	1.67	10.70	11.78	27.30	0.12	5.41
VIT_07s0005g02570	WRKY transcription factor	20.39	34.65	128.83	186.78	26.54	36.92
VIT_16s0050g02510	WRKY transcription factor	15.66	21.43	36.74	103.31	23.85	24.72
VIT_07s0005g01710	WRKY transcription factor	6.56	6.81	7.35	20.64	3.61	3.84
VIT_01s0010g03930	WRKY transcription factor	4.07	22.10	32.43	96.59	5.71	21.80
VIT_17s0000g01280	WRKY transcription factor	0.31	2.62	3.68	10.42	0.44	9.98
VIT_19s0090g00840	WRKY transcription factor	0.44	9.93	4.63	68.05	0.30	2.17
VIT_10s0003g02810	WRKY transcription factor	1.37	25.96	3.77	27.71	1.47	4.30
VIT_01s0026g01730	WRKY transcription factor	1.72	5.47	14.58	27.03	2.9	2.76
VIT_05s0077g00730	WRKY transcription factor	13.33	75.25	26.90	109.90	7.25	22.38
VIT_10s0003g01600	WRKY transcription factor	11.80	47.38	76.53	104.56	17.11	46.80
VIT_02s0025g00420	WRKY transcription factor	0.19	0.46	2.59	13.44	0.62	1.64
VIT_11s0052g01620	Pathogenesis-related protein PR-1	2.35	0.97	0.91	18.12	3.85	7.39
VIT_14s0081g00020	Pathogenesis-related protein PR-1	5.25	17.21	4.56	69.54	0.86	4.87

### Functional verification of the candidate gene *WRKY53*

In higher plants, *WRKY* genes play various roles. Accumulating evidence indicates that WRKY transcription factors are involved in the responses to biotic stresses as well as in plant development [45–48]. The WRKY proteins constitute a large family of transcription factors [44], and some are potentially involved in the regulation of the transcriptional reprogramming responsible for plant immune responses [49]. In the present study, 11 *WRKY* genes were identified as candidate resistance genes from *VID* because their expression was positively correlated with resistance to *C. diplodiella*.

**Fig. 6:**
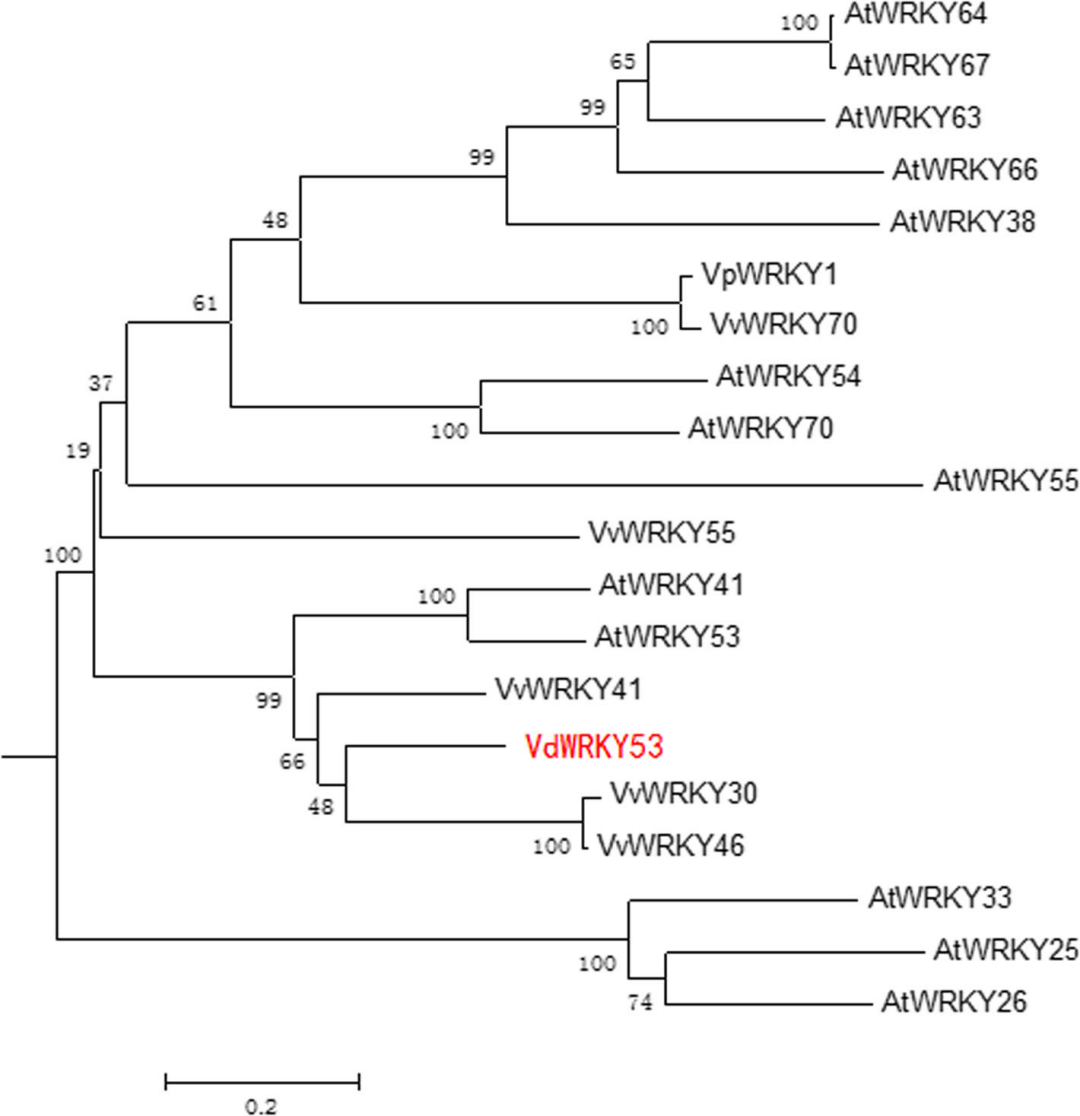
Phylogenetic relationships among the WRKY transcription factors of *Vitis vinifera a*nd Arabidopsis. A phylogenetic tree was constructed using the full-length amino acid sequence (listed in Additional file 4) and neighbor joining (NJ) method in Clustal X version 1.83 and MEGA version 5.0. The scale bar represents 0.2 substitutions per site, and the numbers next to the nodes are bootstrap support values from 1000 replicates

**Fig. 7:**
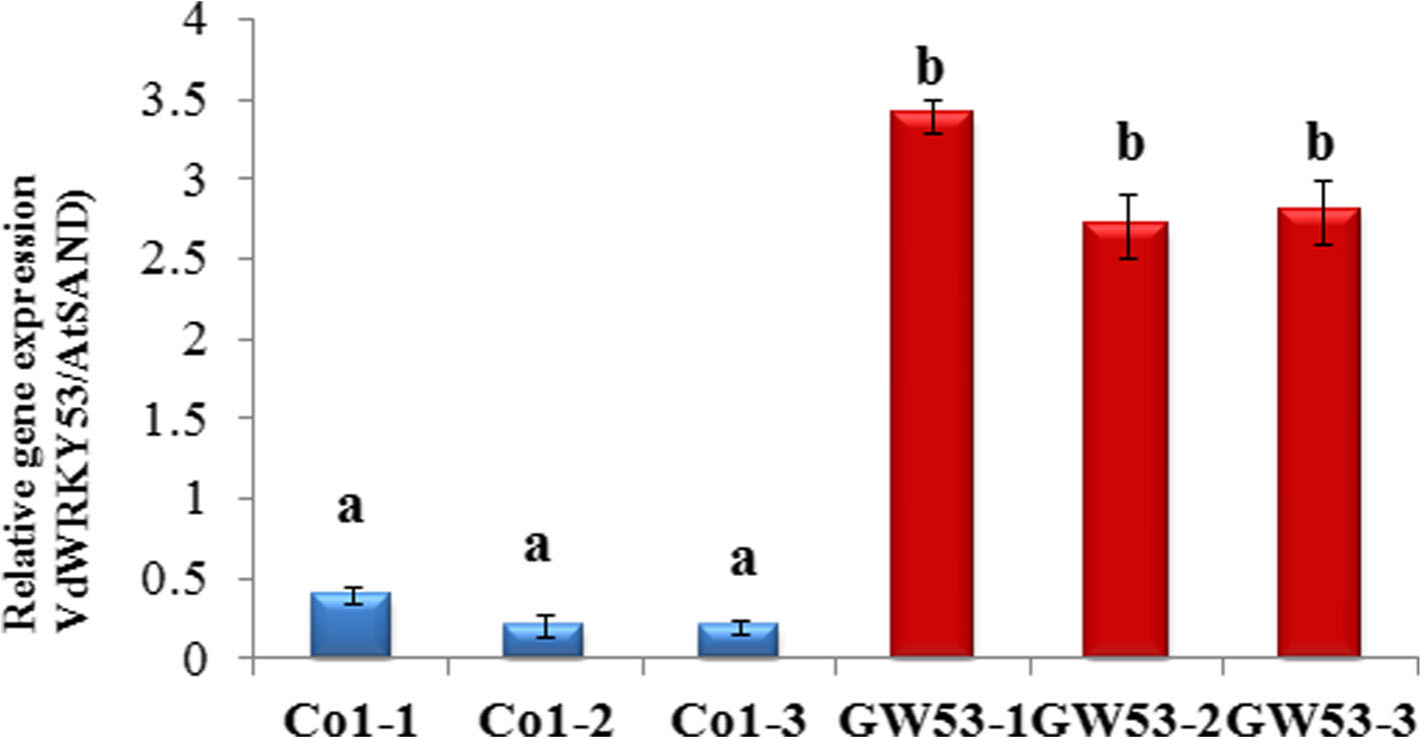
Quantitative real-time PCR analyses of *VdWRKY53* gene expression in wild-type and transgenic Arabidopsis plants. The expression levels of *VdWRKY53* in three wild-type (Col-1, Col-2, and Col-3) and three transgenic (GW53–1, GW53–2, and GW53–3) Arabidopsis lines were analyzed before pathogen infection. The error bars represent the standard deviation of three independent analyses

The gene *VIT_16s0050g02510* (gene ID in the *V. vinifera* Pinot Noir PN40024 reference genome) was selected for further characterization because it was grouped in the subcluster K03 during co-expression analysis and showed a higher expression level in *VID* than in *VIV* at 12 hpi (2.8 fold change; Figs. 4 and 5). The cDNA of this gene was cloned from *VID* and named as *VdWRKY53*. Its cDNA sequence was deposited in GenBank (accession no. KY124243) and it was annotated to WRKY53 based on the grapevine gene nomenclature system [50]. This gene belongs to Group III subfamily of the WRKY family. In plants, the members of this subfamily are considered the most evolutionarily advanced and adaptable and to have coevolved with disease resistance genes [51]. The *VdWRKY53* gene showed higher expression in *VID* than in *VIV* even at 0 hpi. The RPKM value of *VdWRKY53* was 109.9 in response to *C. diplodiella* infection at approximately 12 dpi (Table 4). In the phylogenetic analysis performed here, VdWRKY53 was found to be closely related to VvWRKY30, VvWRKY46, and VvWRKY41 from *V. vinifera* and AtWRKY41 and AtWRKY53 from Arabidopsis (Fig. 6). We produced Arabidopsis transgenic plants by using the pGW53 construct for over-expressing *VdWRKY53* under the control of the CaMV35S promoter. Homozygous transgenic plants were identified from three independent transgenic lines, GW53–1, GW53–2, and GW53–3, by using hygromycin selection and growing them to the T3 generation. Although *VdWRKY53* was expressed in all three transgenic lines, as determined by qRT-PCR, its expression level was approximately three-fold higher than the mRNA level of the Arabidopsis reference gene *AtSAND* (At2g28390; Fig. 7). A background level of expression detected in the wild-type Arabidopsis Col plants could be the result of non-specific amplification of Arabidopsis genes with homology to the qRT-PCR primers used.

**Fig. 8:**
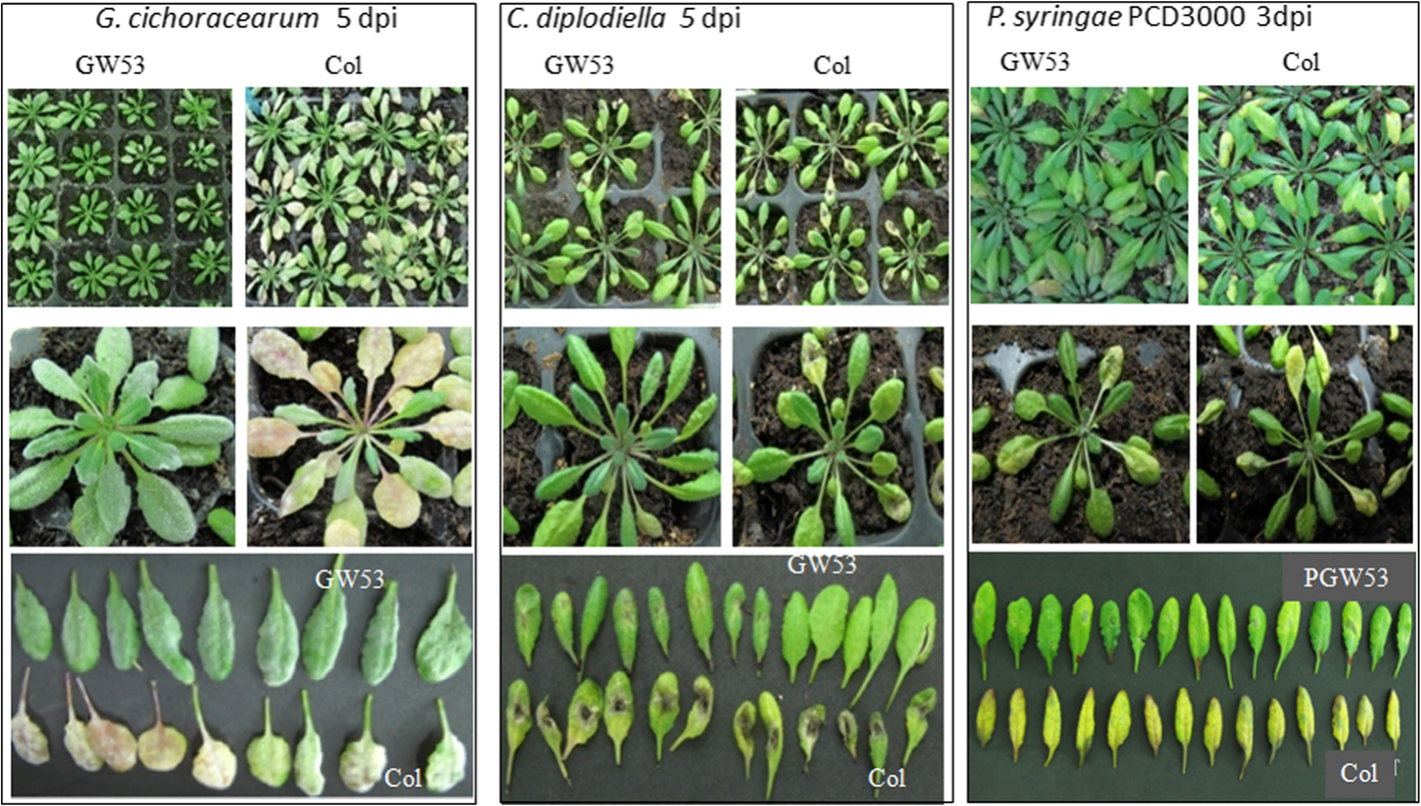
Transgenic Arabidopsis plants over-expressing *VdWRKY53* showed enhanced resistance to pathogens. Homozygous transgenic plants from three different lines were separately infected with *Golovinomyces cichoracearum*, *Coniella diplodiella*, and *Pseudomonas syringae* pv. tomato DC3000. The images show the results of one transgenic line along with wild-type controls post-infection . The plants of other transgenic lines showed similar results

**Fig. 9:**
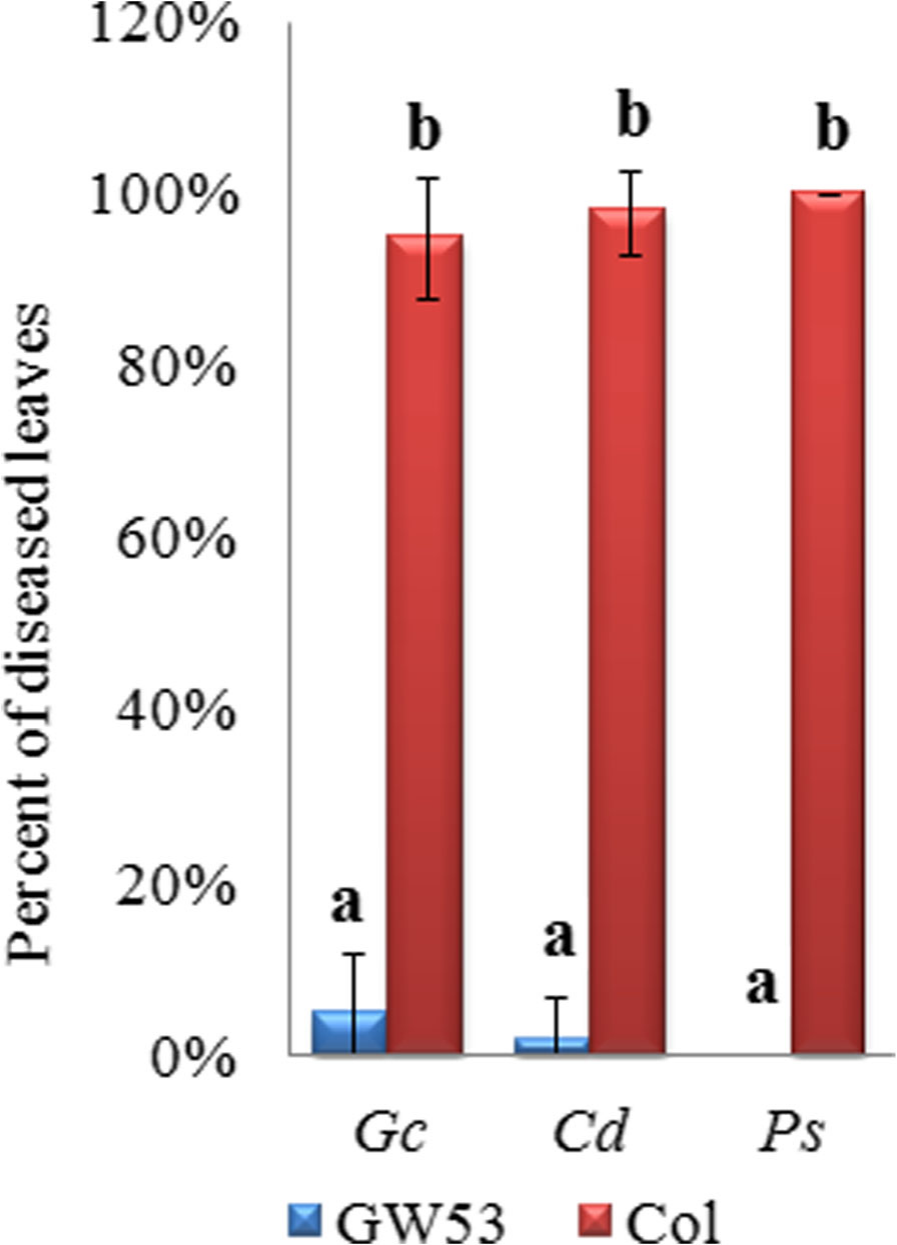
Percentage of Arabidopsis leaves that were free of symptoms after pathogen infection. Homozygous transgenic plants from three different lines were separately infected with *Golovinomyces cichoracearum* (*Gc*), *Coniella diplodiella* (*Cd*), and *Pseudomonas syringae* pv. tomato DC3000 (*Ps*). Disease symptoms were visually examined on 33 leaves for each line at 5 days post-infection (dpi) for *Gc* and *Cd* and at 3 dpi for *Ps*. The error bars represent the standard deviations of three transgenic lines or three populations of wild-type control plants

The homozygous transgenic plants and wild-type Arabidopsis Col control plants were infected with *C. diplodiella*, *G. cichoracearum*, and *PstDC3000*. After infection with *G. cichoracearum*, GW53 plants grew normally with green leaves despite the presence of powdery mildew. However, the wild-type Arabidopsis Col plants developed clear disease symptoms, with yellow and even dead leaves. The same result was noted following *C. diplodiella* and *PstDC3000* infection (Fig. 8). After inoculation with *G. cichoracearum*, *C. diplodiella*, and *PstDC3000*, GW53 plants showed disease symptoms on 5, 3, and 2% of their leaves, respectively, whereas Col plants showed disease symptoms on 95, 97, and 98% of their leaves, respectively (Fig. 9). This indicated that the expression of the grapevine *VdWRKY53* gene in Arabidopsis could improve disease resistance.

## Discussion

Grapevine white rot disease is the most severe threat to *Vitis* species production; in order to identify a germplasm that can be used for research and breeding, we focused on *V. davidii*, which was reported to be a resistant species. We verified the resistance of this germplasm to white rot disease by examining HR lesions using trypan blue staining. The RNA-seq results also showed that more genes were induced in *V. davidii* than in *V. vinifera*, a susceptible species (Fig. 2). However, the expression of important functional genes presented more changes in *VID* than in *VIV*; this finding was similar to that noted in *V. riparia* infected with *Plasmopara viticola*. Further, the same response was found to appear very rapidly after infection in the resistant germplasm *V. riparia* [52]. Co-expression analysis revealed that the K03 cluster included the same resistance pathway as that induced in *V. riparia*; to verify this, we identified the core gene *VdWRKY53*, which was the most up-regulated in the cluster. The over-expression of this gene in Arabidopsis increased its resistance to *C. diplodiella*, *PstDC3000*, and *G. cichoracearum.*

### Rapid response contributes to disease resistance in *VID*

The first step of plant defense against pathogens is the cell wall-associated response. Pathogenic microorganisms need to actively penetrate the plant apoplast for accessing intercellular nutrients. The second step is HR, in which cell death surrounding an infection site restricts the growth of pathogens. Ralph Huckelhoven considered HR-associated cell death to be a complex defense that depends on the timing of HR [45]. In this study, *VID* rapidly showed a typical HR after *C. diplodiella* invasion. The HR and cell death limited pathogen invasion, and then induced the expression of resistance genes in the defense signaling pathways (Fig. 1b, c). The expression of 20 defense-related genes was higher in *VID* than in *VIV* at 0 and 12 hpi (Fig. 3). This result indicated that the expression of defense-related genes in *VID* was a part of the resistance response induced at 12 hpi. This type of response was also observed in other resistant grapevine species such as *V. riparia* after infection with *P. viticola* [38]. This rapid HR response in *VID* could be induced by perceiving microbial molecules or by surveillance of host cellular intactness, which is a common mechanism in plants. This mechanism has also been observed in *V. riparia* infected with *P. viticola* [52]. A defense response was observed in the resistant species *VID*, which rapidly recognized the infection signal and activated HR.

### Candidate genes contributing to defense in *VID*

Detailed pathogen resistance mechanisms have been described in model plant species. They involve complex signaling pathways and a cascade of resistance genes that can be triggered by an elicitor. Plants use pathogen-associated molecular patterns or damage-associated molecular patterns to recognize general elicitors or a special elicitor, which is similar to the innate immune system in animals [53]. When a fungus infects a plant, an exchange of signals occurs between the pathogen and the plant [54].

Cell wall-associated plant defense is the first and most important barrier in basal resistance. Basal resistance seems to be suppressed by virulent pathogens but boosted in induced and race-specific resistance. In *Oryza sativa*, *OsWAK1* transcripts were significantly induced by *Magnaporthe oryzae* and played an important role in rice blast disease resistance [55]. In the present study, two wall-associated receptor kinase (WAK) genes (VIT_18s0041g00020 and VIT_18s0001g11620) were selected as candidate resistance genes.

In plant resistance and animal innate immune systems, serine/threonine-rich repeat receptor-like kinases contribute to the detection of non-self molecules [56, 57]. Five receptor kinase genes were selected as candidates, namely, LRR receptor-like serine/threonine protein kinases (VIT_12s0035g00070, VIT_10s0092g00590, VIT_12s0055g00580, *Vitis_vinifera*_newGene_4892, and *Vitis_vinifera*_newGene_4928). The downstream phosphorylation of a WRKY transcription factor ultimately led to the activation of defense-related genes and the partial restriction of pathogen growth [58].

Eleven WRKY transcription factors responded to fungal infection in the present study, showing a co-expression pattern in the K03 subcluster. The PR proteins are secreted into the defense system during the resistance response. For example, PR-1 has antifungal and antibiotic activity [56, 58–61] and, in the present study, two PR-1 genes (VIT_11s0052g01620 and VIT_14s0081g00020) were shown to participate in the defense against pathogen invasion in *VID*. Plant resistance to diseases might be attributed to specific plant surface and cell wall structures, or HR type self-protection [62]. Our results indicated that the resistance in *VID* was a HR protection, and many candidate genes in the pathogen-associated molecular pattern pathway associated with HR were activated.

### Over-expression of *VdWRKY53* improved disease resistance in Arabidopsis

We cloned the cDNA of *VdWRKY53* and ectopically expressed it in Arabidopsis transgenic plants. As expected, *VdWRKY53* conferred strong resistance to *C. diplodiella*, *PstDC3000*, and *G. cichoracearum* in the transgenic plants. Arabidopsis mutants with loss of *AtWRKY53* function showed delayed development of disease symptoms after infection with *Ralstonia solanacearum* but increased susceptibility toward *PstDC3000* [63]. The expression of *AtWRKY41* is specifically suppressed by a compatible strain of *PstDC3000* in an effector-dependent manner [4]. As VdWRKY53 was classified into the same clade as AtWRKY41 and AtWRKY53, it may play a role in disease resistance. After inoculation with *G. cichoracearum*, *C. diplodiella*, and *PstDC3000*, GW53 plants showed improving disease resistance.

## Conclusions

Innate immune perception triggers both local and systemic responses, allowing a plant to fight off pathogens in a rapid and localized manner and on an extended scale of time and space. The plant defense response to pathogen invasion involves multiple biological processes. Candidate genes from the K03 cluster were co-expressed and classified in the same resistance pathway [41, 49, 64, 65]. Their functions include the recognition of virulence factors, transferring the factor to signaling modules (NB-LRR: the nucleotide binding-leucine rich repeat, NLRs: nucleotide-binding domain and leucine-rich-repeat-containing proteins, and LRR: receptor–like kinase), regulation of switch genes (WRKY) by different modules, and activation of the resistance pathway response. These processes involve genes belonging to the WAK, LRR, WRKY, and PR gene families. In the present study, 20 genes were identified from these families in a co-expression cluster (K03) that showed an expression pattern induced by the infection of grapevine white rot pathogen in *VID* at 12 hpi. The key gene *VdWRKY53* was also found to improve resistance to disease in transgenic Arabidopsis plants. Based on these data, we propose that these 20 candidate genes might contribute to the resistance of *VID* to grapevine white rot disease. As an important germplasm resource, highly resistant to *C. diplodiella, VID* could be used as a parent for resistance breeding in grapevine.

## Additional files

Additional file 1: Primer sequences used in this study. (DOCX 16 kb)

Additional file 2: Number of DEGs between *V. davidii* and *V. vinifera* in different KEGG pathways detected at different infection stages. (DOCX 25 kb)

Additional file 3: Details of the KEGG pathways investigated in this study. (XLSX 122 kb)

Additional file 4: Details of genes classified in subcluster K03. (XLSX 69 kb)

## Abbreviations

COGs: Cluster of Orthologous Groups
GO: Gene Ontology; HR: Hypersensitive response
KEGG: Kyoto Encyclopedia of Genes and Genomes
Nr: Non-redundant proteins and nucleotides
PR-1: pathogenesis-related protein 1
SA: Salicylic acid; UniProt
Swiss: Prot protein
*VI*: *Vitis davidii* accession 0940
*VIV*: *Vitis vinifera* ‘Manicure Finger’
