# Supplementary material for: Identification of the defense-related gene VdWRKY53 from the wild grapevine Vitis davidii using RNA sequencing and ectopic expression analysis in Arabidopsis
Source: Hereditas. 2019 Apr 26;156:14. doi: 10.1186/s41065-019-0089-5 (PMC6486689; doi:10.1186/s41065-019-0089-5)
Supplement: Supplementary file 1 — Primer sequences used in this study. (DOCX 16 kb) [file 41065_2019_89_MOESM1_ESM.docx]

Table S1. Primer sequences used in the study

| No. | Gene ID | Gene name | Primer sequence (Forward) | Primer sequence (Reverse) |
| --- | --- | --- | --- | --- |
| 1 | AF176496 | *VvEF1ｒ* | CAAGAGAAACCATCCCTAGCTG | TCAATCTGTCTAGGAAAGGAAG |
| 2 | At2g28390 | *AtSAND* | AACTCTATGCAGCATTTATCGCCACT | TGATTGCATATCTTTTATCGCCATC |
| 3 | VIT_18s0041g00020 | Wall-associated receptor kinase 2 | GATGAATTGGCTAAGAAGCAGGAG | AGTGACACCCTTAGTCAACATGTCTG |
| 4 | VIT_18s0001g11620 | Wall-associated receptor kinase 2 | TGATCTGTAAAACCCGTCTTCTCC | ACTGGAAATTCACCCACCATGA |
| 5 | VIT_12s0035g00070 | LRR receptor-like serine/  threonine-protein kinase | CACCATGATTGCTCACCTCCTGTA | GGAGCTGTATAACCAAAAGTTCCTGC |
| 6 | VIT_12s0055g00580 | LRR receptor-like serine/  threonine-protein kinase | ATGAAGTTGGTACCCGGAGCTC | TGCATGGTTCCCCACTTGTG |
| 7 | VIT_12s0035g00180 | LRR receptor-like serine/  threonine-protein kinase | ATGCACCATGATTGCTTGCC | CGTAAAAGCAAGTTCTGGAGCAG |
| 8 | Vitis_vinifera  _newGene_4892 | LRR receptor-like serine/  threonine-protein kinase | GAAGTATTCCAGCTGAAATTGGCAA | GCCAGGTTCAGAATTTGGAGTGAT |
| 9 | Vitis_vinifera  _newGene_4928 | LRR receptor-like serine/  threonine-protein kinase | GGTTGAAGCAGCCATGACTGTT | CTCGACCAAGTGAAGAGCCTCTT |
| 10 | VIT_07s0005g02570 | WRKY transcription factor | GGACAAGCCACGCCCTGAAT | TGTTTACGCCTGCTGCAGCC |
| 11 | [VIT_16s0050g02510](http://plants.ensembl.org/Vitis_vinifera/Gene/Summary?db=core;g=VIT_16s0050g02510;r=8:14828036-14830056;tl=n58oycDfj09W5wrX-9604279-177809112) | WRKY transcription factor | ATGGAGAACATGGGAAGTTGG | TGTTGTCCATCATGGAAGGC |
| 12 | VIT_07s0005g01710 | WRKY transcription factor | ATGGAGAGGAGCGGGGTGAT | AGACGGCAGCTGCACCAAAT |
| 13 | VIT_01s0010g03930 | WRKY transcription factor | ATGGATAGCTTCTCCACTCTCTTTCC | GAACTTCCATTGAAGCAGCTGTTG |
| 14 | VIT_17s0000g01280 | WRKY transcription factor | CACCAAATACTTTTTCCCGGTTCAT | GTTTGAAACGCAAATCTGTGCTTTC |
| 15 | VIT_19s0090g00840 | WRKY transcription factor | ATGGACAATGGTGGTAGTAGTGG | TCTCATTGATCCTCCGGTTG |
| 16 | VIT_10s0003g02810 | WRKY transcription factor | GATGAGCCAAGAGATCTCTACTACCATGAC | \| GGTGAAGCTCATGTATGGAGGATCAA \|  \| \| --- \| --- \| |
| 17 | VIT_01s0026g01730 | WRKY transcription factor | ATGGAGGCTGCTGCTTTGGAG | GGCTGATTTGATCCGATCCTCC |
| 18 | VIT_05s0077g00730 | WRKY transcription factor | ATGGAGGAGAAAGAGCAAGTGAAGAC | GGAGGCTGAAGCAAATCGAATAGA |
| 19 | VIT_10s0003g01600 | WRKY transcription factor | ATGGACGGCAGATTCAACAGTAAC | TCTTTTGCATGGCTCGCCTA |
| 20 | VIT_02s0025g00420 | WRKY transcription factor | ACTGGGATTTGCAGTCTGTGGTT | GCTTGTACAACTGCTCCAATTCGT |
| 21 | VIT_11s0052g01620 | Pathogenesis-related protein PR-1 | ATGAGTCCATGGCACTGTGTTCTTG | ATAGTGCGCCAGCTTGGAATCC |
| 22 | VIT_14s0081g00020 | Pathogenesis-related protein PR-1 | AACTACCTGTGGCAGGTGCCTTAG | GCAAGATAACAGTGCACGAACTGG |
