# Supplementary material for: Identification of the defense-related gene VdWRKY53 from the wild grapevine Vitis davidii using RNA sequencing and ectopic expression analysis in Arabidopsis
Source: Hereditas. 2019 Apr 26;156:14. doi: 10.1186/s41065-019-0089-5 (PMC6486689; doi:10.1186/s41065-019-0089-5)
Supplement: Supplementary file 2 — Number of DEGs between V. davidii and V. vinifera in different KEGG pathways detected at different infection stages. (DOCX 25 kb) [file 41065_2019_89_MOESM2_ESM.docx]

Table S2. **Number of DEGs between *V. davidii* and *V. vinifera* in different KEGG pathways detected at different infecting stages**

|  | **KEGG pathways** | **Vd2/ Vd1** | **Vd3/ Vd1** | **MF2/**  **MF1** | **MF3/**  **MF1** | **Total number** |
| --- | --- | --- | --- | --- | --- | --- |
| 1 | 2-Oxocarboxylic acid metabolism | 8 | 10 | 11 | 12 | 47 |
| 2 | ABC transporters | 3 | 2 | 2 | 1 | 21 |
| 3 | Alanine, aspartate and glutamate metabolism | 12 | 13 | 9 | 13 | 41 |
| 4 | alpha-Linolenic acid metabolism | 11 | 6 | 6 | 4 | 31 |
| 5 | Amino sugar and nucleotide sugar metabolism | 18 | 13 | 12 | 11 | 101 |
| 6 | Aminoacyl-tRNA biosynthesis | 5 | 4 | 5 | 4 | 108 |
| 7 | Arachidonic acid metabolism | 3 | 3 | 3 | 2 | 11 |
| 8 | Arginine and proline metabolism | 14 | 15 | 14 | 16 | 62 |
| 9 | Ascorbate and aldarate metabolism | 10 | 7 | 12 | 7 | 30 |
| 10 | Basal transcription factors | 0 | 1 | 1 | 0 | 41 |
| 11 | Base excision repair | 2 | 3 | 3 | 4 | 34 |
| 12 | beta-Alanine metabolism | 7 | 5 | 4 | 5 | 36 |
| 13 | Biosynthesis of amino acids | 41 | 42 | 39 | 46 | 202 |
| 14 | Biosynthesis of secondary metabolites | 166 | 154 | 130 | 132 | 791 |
| 15 | Biosynthesis of unsaturated fatty acids | 8 | 6 | 8 | 6 | 26 |
| 16 | Brassinosteroid biosynthesis | 0 | 0 | 0 | 0 | 9 |
| 17 | Biotin metabolism | 2 | 3 | 3 | 5 | 16 |
| 18 | Brassinosteroid biosynthesis | 2 | 1 | 1 | 0 | 99 |
| 19 | Butanoate metabolism | 3 | 3 | 3 | 5 | 25 |
| 20 | C5-Branched dibasic acid metabolism | 1 | 1 | 2 | 1 | 7 |
| 21 | Carbon fixation in photosynthetic organisms | 25 | 22 | 23 | 21 | 60 |
| 22 | Carbon metabolism | 51 | 44 | 41 | 42 | 188 |
| 23 | Carotenoid biosynthesis | 10 | 9 | 11 | 9 | 28 |
| 24 | Cutin, suberine and wax biosynthesis | 0 | 0 | 0 | 0 | 13 |
| 25 | Circadian rhythm - plant | 11 | 9 | 9 | 11 | 29 |
| 26 | Citrate cycle (TCA cycle) | 8 | 10 | 8 | 7 | 48 |
| 27 | Cutin, suberine and wax biosynthesis | 1 | 2 | 0 | 0 | 13 |
| 28 | Cyanoamino acid metabolism | 3 | 4 | 4 | 6 | 40 |
| 29 | Cysteine and methionine metabolism | 16 | 13 | 11 | 15 | 72 |
| 30 | Degradation of aromatic compounds | 5 | 0 | 1 | 0 | 15 |
| 31 | Diterpenoid biosynthesis | 2 | 2 | 0 | 0 | 14 |
| 32 | DNAreplication | 1 | 1 | 0 | 1 | 43 |
| 33 | Endocytosis | 8 | 5 | 6 | 1 | 76 |
| 34 | Etherlipidmetabolism | 2 | 1 | 2 | 1 | 20 |
| 35 | Fattyacidbiosynthesis | 4 | 4 | 3 | 5 | 31 |
| 36 | Fattyaciddegradation | 12 | 5 | 6 | 6 | 38 |
| 37 | Fattyacidelongation | 3 | 5 | 4 | 4 | 24 |
| 38 | Fattyacidmetabolism | 11 | 11 | 11 | 13 | 62 |
| 39 | Flavonoidbiosynthesis | 12 | 8 | 8 | 3 | 33 |
| 40 | Folatebiosynthesis | 2 | 1 | 1 | 2 | 16 |
| 41 | Fructoseandmannosemetabolism | 12 | 7 | 7 | 7 | 44 |
| 42 | Galactosemetabolism | 8 | 6 | 4 | 8 | 40 |
| 43 | Glutathionemetabolism | 13 | 16 | 13 | 11 | 76 |
| 44 | Glycerolipidmetabolism | 15 | 11 | 12 | 10 | 46 |
| 45 | Glycerophospholipidmetabolism | 9 | 8 | 7 | 6 | 67 |
| 46 | Glycine,serineandthreoninemetabolism | 17 | 16 | 18 | 17 | 50 |
| 47 | Glycolysis/Gluconeogenesis | 32 | 17 | 18 | 17 | 108 |
| 48 | Glycosaminoglycandegradation | 2 | 1 | 0 | 0 | 10 |
| 49 | Glycosphingolipidbiosynthesis  -globoseries | 2 | 1 | 2 | 2 | 9 |
| 50 | Glycosylphosphatidylinositol(GPI)-anchorbiosynthesis | 2 | 1 | 1 | 0 | 22 |
| 51 | Glyoxylateanddicarboxylatemetabolism | 19 | 16 | 21 | 16 | 54 |
| 52 | Histidinemetabolism | 5 | 3 | 3 | 4 | 17 |
| 53 | Homologousrecombination | 1 | 1 | 1 | 2 | 43 |
| 54 | Inositolphosphatemetabolism | 6 | 7 | 9 | 6 | 43 |
| 55 | Isoquinolinealkaloidbiosynthesis | 3 | 5 | 3 | 4 | 20 |
| 56 | Limoneneandpinenedegradation | 5 | 3 | 4 | 2 | 9 |
| 57 | Linoleicacidmetabolism | 7 | 2 | 2 | 2 | 12 |
| 58 | Lysinebiosynthesis | 2 | 4 | 2 | 5 | 13 |
| 59 | Lysinedegradation | 5 | 3 | 4 | 2 | 25 |
| 60 | Metabolicpathways | 287 | 249 | 236 | 219 | 1497 |
| 61 | mRNAsurveillancepathway | 8 | 8 | 5 | 9 | 96 |
| 62 | N-Glycanbiosynthesis | 2 | 2 | 3 | 1 | 41 |
| 63 | Nicotinateandnicotinamidemetabolism | 2 | 2 | 0 | 1 | 13 |
| 64 | Nitrogenmetabolism | 10 | 8 | 9 | 8 | 30 |
| 65 | Non-homologousend-joining | 0 | 0 | 1 | 0 | 8 |
| 66 | Nucleotideexcisionrepair | 3 | 2 | 0 | 0 | 52 |
| 67 | Onecarbonpoolbyfolate | 3 | 2 | 3 | 1 | 16 |
| 68 | Nucleotideexcisionrepair | 0 | 0 | 0 | 1 | 52 |
| 69 | Otherglycandegradation | 5 | 2 | 5 | 3 | 17 |
| 70 | Oxidativephosphorylation | 10 | 14 | 8 | 7 | 146 |
| 71 | Onecarbonpoolbyfolate | 0 | 0 | 0 | 3 | 16 |
| 72 | PantothenateandCoAbiosynthesis | 2 | 3 | 3 | 3 | 23 |
| 73 | Pentoseandglucuronateinterconversions | 13 | 9 | 9 | 8 | 53 |
| 74 | Pentosephosphatepathway | 13 | 10 | 9 | 12 | 42 |
| 75 | Peroxisome | 14 | 17 | 13 | 14 | 63 |
| 76 | Phagosome | 3 | 6 | 7 | 11 | 71 |
| 77 | Phenylalaninemetabolism | 15 | 10 | 10 | 9 | 93 |
| 78 | Phenylalanine,tyrosineandtryptophanbiosynthesis | 1 | 6 | 4 | 6 | 46 |
| 79 | Phenylpropanoidbiosynthesis | 19 | 16 | 12 | 12 | 118 |
| 80 | Phosphatidylinositolsignalingsystem | 4 | 4 | 6 | 3 | 35 |
| 81 | Photosynthesis | 30 | 11 | 29 | 7 | 82 |
| 82 | Photosynthesis-antennaproteins | 17 | 9 | 17 | 8 | 18 |
| 83 | Planthormonesignaltransduction | 30 | 24 | 36 | 25 | 236 |
| 84 | Plant-pathogeninteraction | 24 | 23 | 20 | 21 | 105 |
| 85 | Porphyrinandchlorophyllmetabolism | 9 | 12 | 10 | 9 | 41 |
| 86 | Propanoatemetabolism | 7 | 3 | 3 | 3 | 36 |
| 87 | Proteinexport | 1 | 1 | 2 | 2 | 41 |
| 88 | Proteinprocessinginendoplasmicreticulum | 19 | 24 | 13 | 21 | 186 |
| 89 | Purinemetabolism | 14 | 15 | 8 | 15 | 128 |
| 90 | Pyrimidinemetabolism | 10 | 15 | 7 | 13 | 101 |
| 91 | Pyruvatemetabolism | 19 | 15 | 14 | 13 | 77 |
| 92 | Riboflavinmetabolism | 1 | 0 | 1 | 0 | 8 |
| 93 | Ribosome | 10 | 40 | 13 | 13 | 295 |
| 94 | Ribosomebiogenesisineukaryotes | 13 | 14 | 11 | 9 | 83 |
| 95 | RNAdegradation | 17 | 8 | 9 | 10 | 87 |
| 96 | RNApolymerase | 2 | 5 | 3 | 4 | 41 |
| 97 | RNAtransport | 17 | 13 | 7 | 8 | 136 |
| 98 | Selenocompoundmetabolism | 2 | 1 | 1 | 2 | 14 |
| 99 | Sesquiterpenoidandtriterpenoidbiosynthesis | 2 | 1 | 2 | 1 | 8 |
| 100 | Sphingolipidmetabolism | 6 | 1 | 3 | 3 | 25 |
| 101 | Spliceosome | 5 | 14 | 3 | 3 | 149 |
| 102 | Starchandsucrosemetabolism | 30 | 29 | 25 | 27 | 141 |
| 103 | Steroidbiosynthesis | 4 | 2 | 1 | 3 | 22 |
| 104 | Stilbenoid,diarylheptanoidandgingerolbiosynthesis | 6 | 5 | 1 | 3 | 18 |
| 105 | Sulfurmetabolism | 6 | 4 | 5 | 8 | 29 |
| 106 | Sulfurrelaysystem | 0 | 1 | 0 | 2 | 12 |
| 107 | Stilbenoid,diarylheptanoidandgingerolbiosynthesis | 6 | 5 | 7 | 1 | 18 |
| 108 | Synthesisanddegradationofketonebodies | 1 | 1 | 1 | 1 | 5 |
| 109 | Taurineandhypotaurinemetabolism | 3 | 2 | 1 | 1 | 11 |
| 110 | Terpenoidbackbonebiosynthesis | 8 | 10 | 8 | 8 | 45 |
| 111 | Thiaminemetabolism | 3 | 3 | 2 | 1 | 14 |
| 112 | Tropane,piperidineandpyridinealkaloidbiosynthesis | 4 | 6 | 6 | 5 | 23 |
| 113 | Tryptophanmetabolism | 6 | 4 | 5 | 4 | 29 |
| 114 | Tyrosinemetabolism | 7 | 5 | 5 | 6 | 42 |
| 115 | Ubiquinoneandotherterpenoid-quinonebiosynthesis | 7 | 7 | 3 | 5 | 27 |
| 116 | Ubiquitinmediatedproteolysis | 15 | 10 | 13 | 9 | 105 |
| 117 | Valine,leucineandisoleucinebiosynthesis | 4 | 4 | 4 | 4 | 16 |
| 118 | Valine,leucineandisoleucinedegradation | 8 | 7 | 6 | 6 | 42 |
| 119 | VitaminB6metabolism | 2 | 1 | 1 | 1 | 9 |
| 120 | Zeatinbiosynthesis | 3 | 2 | 1 | 3 | 17 |
